# Longitudinal Cross-Lagged Analysis Between Depressive Symptoms, Social Withdrawal, Self-Esteem, and School Adaptation in Multicultural Adolescents

**DOI:** 10.5334/pb.1310

**Published:** 2025-01-20

**Authors:** Chung Choe, Seunghee Yu

**Affiliations:** 1 Department of Economics, Konkuk University, Seoul, South Korea; 2 College of General Education, Kookmin University, Seoul, South Korea

**Keywords:** multicultural adolescents, depressive symptoms, social withdrawal, self-esteem, school adaptation

## Abstract

In Korea, as the number of multicultural families formed through the marriage of Korean men and foreign women from lower-income countries such as China, Southeast Asia, and Central Asia increases, the psychosocial adaptation of adolescents from these families is becoming increasingly important. This study examines the longitudinal and reciprocal relationships among depressive symptoms, social withdrawal, self-esteem, and school adaptation in multicultural adolescents in high schools. We applied an autoregressive cross-lagged model to a sample of 594 multicultural adolescents extracted from three consecutive years of data from the Multicultural Adolescents Panel Survey and found that depressive symptoms and school adaptation had reciprocal negative relationships in multicultural adolescents’ first and second years of high school. In these years, while higher school adaptation led to lower social withdrawal, social withdrawal did not affect school adaptation. In the second and third years of high school, self-esteem and school adaptation had a reciprocal positive relationship. Females had more severe psycho-emotional problems than males. Child neglect increased the risk of depressive symptoms and social withdrawal while lowering self-esteem. A high household income was associated with lower social withdrawal. Adolescents in urban areas exhibited higher levels of depressive symptoms, lower self-esteem, and poorer school adaptation compared to those in rural areas. These results imply that reducing parents’ neglect of their children is necessary to alleviate depressive symptoms and school maladaptation among multicultural adolescents. Moreover, financial support for multicultural adolescents in their learning can improve school maladaptation and reduce social withdrawal.

## Introduction

In Korea, the formation of multicultural families primarily occurs through unions between Korean men and women from lower-income countries such as China, Southeast Asia, and Central Asia (Kim, 2022). These women initially come to Korea on a marriage immigration visa and acquire permanent residency or citizenship after residing in Korea for a certain period. This trend has been growing since the 1990s alongside an increase in the influx of foreigners into Korea. Consequently, there has been a rise in the number of multicultural families established through these international marriages. According to data from Statistics Korea (2022), there were 15,341 cross-cultural marriages in Korea in 2020, constituting 7% of all marriages. Notably, 72% of these marriages were between Korean men and foreign women.

Also in 2020, there were 16,421 births in multicultural families in Korea, comprising 6% of the total births that year, as reported by Statistics Korea (2022). The Ministry of Education observed a contrasting trend in the same year: while the total number of adolescents in middle and high schools had decreased by 556,000 over the five years before 2020, the population of adolescents from multicultural backgrounds had increased by 14,455. This led to a continuous rise in the proportion of multicultural adolescents within the overall adolescent population. By 2020, there were 39,251 adolescents from multicultural families, representing 1.5% of all adolescents (Korean Ministry of Education, 2020b).

Adolescents, referring to teenagers of middle and high school age, face numerous developmental challenges, such as establishing their identity, nurturing social connections, preparing for further education, and exploring career options (Heo, 2012; Oh, 2016). In Korea, adolescents often endure significant psychosocial issues like depression, social withdrawal, low self-esteem, and difficulty adapting to school, which are largely attributed to the intense pressure of academic achievement and career-related concerns (An et al., 2013; Jeon et al., 2019). In addition, the COVID-19 pandemic and the associated sanitary measures have led to an increase in negative mental health indicators among adolescents worldwide (Marques de Miranda et al., 2020). This trend can be attributed to the pandemic itself and the resulting restrictions on social interactions, which are critical for this age group. Social isolation due to restrictions on social gatherings, school closures, and lockdowns as well as reduced physical activities and increased screen time and social media use have exacerbated adolescents’ depression, lowered their self-esteem, and increased their social withdrawal (Cruz et al., 2023; Sikorska et al., 2021). Moreover, the challenges of adapting to remote education including technical issues; a lack of direct support from teachers; limited access to resources; difficulties in maintaining concentration, understanding material, and staying motivated; and reduced extracurricular activities have contributed to increased negative indicators of school adaptation (Salmela-Aro et al., 2021).

Significantly, adolescents from multicultural families (hereinafter referred to as multicultural adolescents) are at a higher risk of these psychosocial challenges compared to their peers from non-multicultural families. Factors contributing to this increased vulnerability include stress from cultural adaptation, identity confusion, experiences of discrimination and exclusion, and challenges in building friendships, communication barriers with foreign-born parents (Oh, 2016). Because foreign mothers often lack proficiency in Korean and children do not systematically learn their mothers’ languages, children from multicultural families in Korea, especially teenagers, frequently do not speak their mothers’ languages fluently and prefer to speak Korean at home.

Multicultural adolescents encounter difficulties such as struggling academically, leaving school early, and facing barriers in career exploration. These issues often stem from a lack of parental engagement and support with school activities, limited financial resources, and an absence of adequate information or support structures (Jeon et al., 2019; Lee, 2020a). Data from the Korean Ministry of Education (2020a) reveal that in 2018, the dropout rate among multicultural adolescents in middle (1.34%) and high school (1.91%) exceeded the overall dropout rates for adolescents (middle school: 0.73%, high school: 1.62%). Notably, the dropout rate for multicultural adolescents was significantly higher in high school than in middle school.

High school students experience greater stress compared to their elementary and middle school counterparts, primarily due to academic pressure, which has been shown to adversely impact their mental well-being (Yeom et al., 2020). Therefore, understanding and addressing the psychosocial issues faced by multicultural adolescents in high school is crucial. With these points in mind, this study aims to explore how depressive symptoms, social withdrawal, self-esteem, and school adaptation interrelate in multicultural adolescents from families with a foreign mother and a Korean father at the high school level. Furthermore, we aim to identify factors that influence depressive symptoms and social withdrawal, as outcomes of adolescents’ psycho-emotional state, as well as self-esteem and school adaptation.

## Literature Review

### Depressive Symptoms, Social Withdrawal, and Self-Esteem in Multicultural Adolescents

Multicultural families formed through unions between native men and foreign women from lower-income countries such as China, Southeast Asia, and Central Asia are a unique family structure primarily found in Korea, Taiwan, and Japan (Kim, 2022; Lin et al., 2011). This literature review mainly pertains to the context of these Asian countries. Children from marital-immigrant families often experience higher levels of depression compared to those from native families (Jang & Park, 2019; Lin et al., 2011). Lin et al. (2011) examined depression levels in elementary school children from both native and marital immigrant families in Taiwan using a logistic regression model and revealed that family- and school-related factors had a stronger impact on children from marital-immigrant families than on those from native families. For these children, a strong bond with parents, a harmonious family environment, and good relationships with peers were found to lower the risk of depression; conversely, poor academic performance was linked to an increased risk of depression.

In a similar vein, in a study by Jang and Park (2019), multiple regression results showed that among middle school students from multicultural families in Korea, depressive symptoms decreased when their mothers exhibited more democratic,^1^ independent, affectionate, and receptive parenting behaviors. While academic stress was linked to an increase in depressive symptoms, stronger peer attachments and higher self-esteem were associated with a reduction in these symptoms. Oh (2016) conducted a multiple regression analysis on depressive symptoms in Korean multicultural adolescents aged 9 to 24 years and found that females exhibited higher levels of depressive symptoms than males and that these symptoms intensified with age. However, a better family relationship and higher self-esteem were correlated with lower depressive symptoms in these adolescents.

Social withdrawal is a tendency to be shy, passive, and avoidant in interactions with people (Rubin et al., 2009). It is a kind of coping strategy for social situations, which leads adolescents to become isolated from social situations (Oh, 2018). Socially withdrawn adolescents are more likely to be less socially competent (Bohlin et al., 2005) and experience peer neglect and rejection than typical adolescents (Deater-Deckard, 2001). Social withdrawal is linked to anxiety, depression, and school maladaptation (Jeong, 2016; Rubin et al., 2009).

The experience of adverse treatment can lead to social withdrawal (Link & Phelan, 2001). In Korean society, compared to native adolescents, multicultural adolescents are more exposed to cultural prejudice, discrimination, and situations where others are negatively evaluating them. This adverse treatment occurs because these children differ from others with Korean parents in cultural characteristics, socioeconomic status,^2^ appearance, etc., and these experiences can make them socially withdrawn (Jo & Oh, 2020). As multicultural adolescents age, social withdrawal tends to increase (Oh, 2019).

The social withdrawal of multicultural adolescents in Korea differs according to gender, school-related factors (e.g., friend relationships, teacher relationships), parenting style, and the family’s socioeconomic status. For example, Jang et al. (2020) longitudinally analyzed changes in social withdrawal in multicultural adolescents using a growth mixture model and found that, on average, social withdrawal was higher in female versus male adolescents, and this trend continued throughout adolescence. In addition, multiple regression (Mo, 2018) and path (Jo & Oh, 2020; Oh, 2018) analyses of cross-sectional data have demonstrated that as the relationship with teachers and friends improves, social withdrawal decreases. Oh’s (2019) structural equation model revealed that child neglect increased social withdrawal, and Hong and Park’s (2017) multivariate latent growth model indicated that adolescents who experienced child neglect exhibited a greater increase in social withdrawal over time compared to adolescents who did not. By contrast, Lee’s (2010) multiple regression analysis of social withdrawal in children from multicultural families found that the family’s high socioeconomic status decreased these children’s social withdrawal.

Regression analyses using cross-sectional data have also found that higher academic achievement is associated with the higher economic status of parents (Bong & Kim, 2017; Lee & Lee, 2019) and higher self-esteem. Moreover, while children of parents who raise their children democratically have higher self-esteem, a neglectful parenting attitude is associated with children’s lower self-esteem (Lee, 2020b). Regarding multicultural adolescents, No et al. (2019) analyzed the longitudinal structural relationship among appearance, self-esteem, and school adjustment and found that sixth-grade adolescents who reported higher satisfaction with their appearance had higher self-esteem in seventh grade, and the higher their self-esteem in seventh grade, the better they adjusted to school in eighth grade. A study analyzing the impact of child neglect on multicultural adolescents’ self-esteem using the multivariate latent growth model (Oh, 2019) determined that self-esteem decreased with increasing school grades. In addition, as the average value of child neglect increased, the average value of self-esteem decreased, and as the increase rate of child neglect increased over time, the increase rate of self-esteem decreased.

Given that urbanization may increase the risk of psychosocial issues such as depression, social withdrawal, low self-esteem, and school maladaptation (Elgar, et al., 2003), the level of these psychosocial issues may differ between multicultural adolescents living in urban versus rural areas. Indeed, some studies have shown that a rural environment can help counteract the development of depression, acculturation stress, and problem behaviors (Dobbins et al., 2022; Yilmaz et al., 2022). Although multicultural adolescents living in cities can access a variety of resources and support networks, the high stress and competitive environment can lead to mental health problems and maladaptation (Elgar, et al., 2003). In rural areas, because communities are often smaller and closer-knit, multicultural adolescents can form strong social bonds (Biggar et al., 2016). However, they may experience a lack of educational resources, services, and facilities (Sjolie & Thuen, 2002).

Multicultural adolescents tend to experience increased depressive symptoms and social withdrawal and decreased self-esteem over time, suggesting that their psychosocial problems may be greater during high school (Oh, 2016; Oh, 2019). However, existing studies analyzing the psychosocial issues of children from multicultural families have mainly targeted multicultural adolescents in early or middle adolescence, such as children or middle school students (Lin et al., 2011; Jang & Park, 2019; Jo & Oh, 2020). Although some studies have conducted longitudinal analyses of the psychosocial problems of multicultural adolescents in elementary or middle school (Jang et al, 2020; Oh, 2019), studies addressing the psychosocial problems of multicultural adolescents in high school using longitudinal data are scarce.

### School Adaptation and Depressive Symptoms, Social Withdrawal, and Self-Esteem in Multicultural Adolescents

School adaptation is one of the most important outcomes of a successful acculturation process for adolescents with an immigrant background (Schachner et al., 2014). School is where multicultural adolescents not only learn about Korean values, beliefs, and behaviors but also adapt to cultural and social expectations (Portes & Rumbaut, 2001). As such, schools are institutions of education and socialization, so school adaptation is crucial for multicultural adolescents to adapt well to Korean society and grow into healthy community members.

School adaptation of adolescents with immigrant backgrounds is closely related to their demographic characteristics and psycho-emotional state (Bae & Lee, 2014; Jeon & Yoon, 2013; Rumbaut, 2005). Immigrant boys generally show higher school dropout rates and lower academic achievement than immigrant girls (Rumbaut, 2005). High socioeconomic status, good parental relationships, and parental support increase the school adaptation of immigrant adolescents (Bhattacharya, 2000; Rumbaut, 2005).

Additionally, while multicultural adolescents’ self-esteem positively affects their school adaptation (Jeon & Yoon, 2013; Kim, 2019), depressive symptoms and social withdrawal negatively impact it (Kim, 2020; Yu & Kim, 2018). On the other hand, multicultural adolescents’ school adaptation has also been considered a significant variable influencing their psycho-emotional state. For example, an amicable relationship with teachers—one area of school adaptation—is a factor that reduces multicultural adolescents’ depressive symptoms and social withdrawal (Jo & Oh, 2020). Social withdrawal, depression, and anxiety also decreased as students were more satisfied with school life (Bae & Lee, 2014). Similarly, students’ higher self-esteem is positively associated with a higher sense of academic efficacy (Chun, 2018), a better relationship with teachers and peers, and higher satisfaction with school life (Kim, 2012).

### Current Study

The relationship between school adaptation and psycho-emotional state—depressive symptoms, social withdrawal, and self-esteem—among multicultural adolescents is likely to be bidirectional. However, because existing research has used cross-sectional data to establish one-way relationships between these variables, it has not been able to determine any reciprocal or causal relationship between them. Longitudinal analysis can compensate for some of these limitations by establishing the relationship between independent variables that precede in time and dependent variables that occur later (Raudenbush, 2001).

Given these points, using longitudinal data from the Multicultural Adolescents Panel Survey (MAPS), this study analyzes the reciprocal relationships between the psycho-emotional state (depressive symptoms, social withdrawal, and self-esteem) and school adaptation of high school multicultural adolescents. Previous studies have implied that gender (Mo, 2018; Oh, 2016) and residence area (Dobbins et al., 2022; Elgar, et al., 2003; Yilmaz et al., 2022), among demographic characteristics, and household income (Bong & Kim, 2017) and child neglect (Jang & Park, 2019; Lee, 2020b; Lin et al., 2011; Oh, 2016; Oh, 2019), among family-related conditions, are important factors that can affect multicultural adolescents’ psycho-emotional state and school adaptation.

We applied an autoregressive cross-lagged model to examine 1) changes in depressive symptoms, social withdrawal, self-esteem, and school adaptation over time (autoregressive effects); 2) the effect of depressive symptoms, social withdrawal, and self-esteem on school adaptation over time (cross-lagged effects); 3) the effect of school adaptation on depressive symptoms, social withdrawal, and self-esteem over time (cross-lagged effects); and 4) the effects of the sociodemographic characteristics of gender, household income, child neglect, and area on multicultural adolescents’ depressive symptoms, social withdrawal, self-esteem, and school adaptation. These variables are assumed to moderate the longitudinal relationships between depressive symptoms, social withdrawal, self-esteem, and school adaptation.

## Method

### Data and Sample

The data for this study were extracted from three waves (2017 to 2019) of the Multicultural Adolescents Panel Survey (MAPS) conducted by the National Youth Policy Institute (NYPI). In 2011, a stratified, multistage cluster sampling method was employed to select a sample of multicultural adolescents among fourth-grade students in 2,537 elementary schools across 16 cities and provinces. MAPS, an annual survey initiated in 2011, focuses on various aspects of multicultural youth life, including school experiences, psychosocial adjustment, physical development, and dynamics in parent–child relationships. After recruiting households through schools where multicultural students were attending, investigators visited the homes of parents and students who agreed to the survey and conducted interviews. The mother, who was not proficient in Korean, conducted the interview using a foreign-language questionnaire. Computer Assisted Personal Interviewing was the survey tool used from the first to the seventh waves, and Tablet Assisted Personal Interviewing was used from the ninth wave onward. The anonymity and privacy of the participants were ensured when collecting the data. Because we used secondary data, the approval of the Research Ethics Committee was not required. The data are available on the NYPI website (www.nypi.re.kr) upon request.

In the seventh wave (2017), 1,260 adolescents completed the survey, but attrition occurred in the survey process in the following years, resulting in 1,197 adolescents for Wave 8 and 1,146 for Wave 9. We performed the missing completely at random (MCAR) test and confirmed that the data achieve the MCAR level, i.e., the *p* value is larger than 0.05; thus, we can accept the null hypothesis that the data were missing completely at random (Little, 1988). We removed cases with missing values. The specific data used in this study, taken from the seventh, eighth, and ninth waves of MAPS, align with the participants’ first, second, and third years of high school, respectively. Our research focused on 594 adolescents from multicultural families who participated in all three waves from 2017 to 2019 and have a Korean father and a foreign mother who is from China (43.6%), Vietnam (3.9%), the Philippines (45.3%), or Thailand (7.2%). The sample consists of 49.5% boys and 50.5% girls. Approximately 35% of the adolescents live in rural areas, while 65% live in urban areas. The average age was about 16 years in Wave 7, 17 years in Wave 8, and 18 years in Wave 9.

### Measures

**Figure 1:**
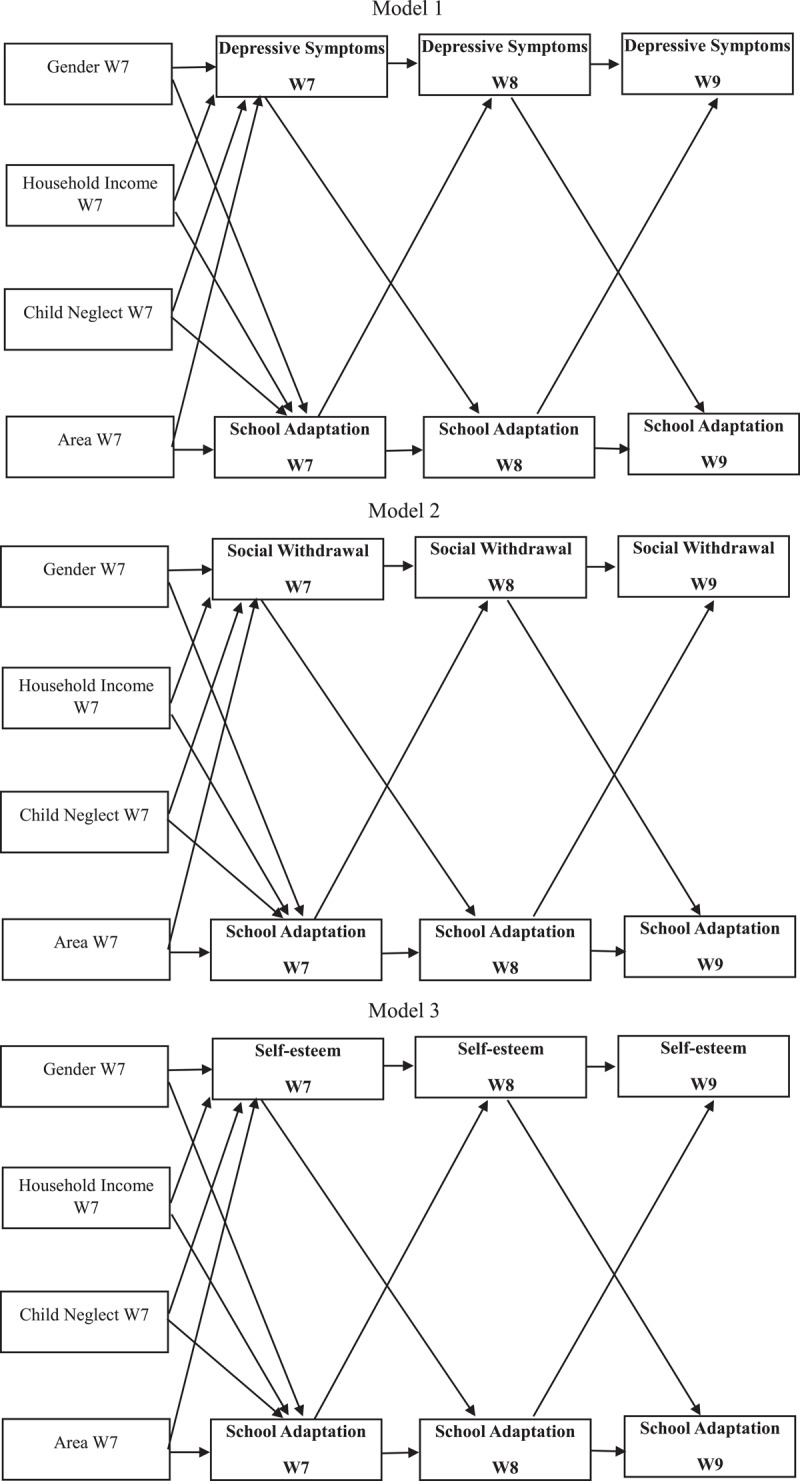
Autoregressive cross-lagged research models. *Note*. W7 = Wave 7; W8 = Wave 8; W9 = Wave 9.

Figure 1 presents this study’s model. All measures were self-reported by the adolescents. School adaptation was measured with four items (Cronbach’s α: W7 = 0.916, W8 = 0.920, W9 = 0.918) from the peer relationship scale (Kim, 2013), four items (Cronbach’s α: W7 = 0.862, W8 = 0.865, W9 = 0.885) from the academic scale (Kim, 2013), and three items (Cronbach’s α: W7 = 0.888, W8 = 0.895, W9 = 0.878) from a scale measuring relationships with teachers (Hwang & Kim, 2006). The Cronbach’s alpha value of all items measuring school adaptation was 0.898 in Wave 7, 0.905 in Wave 8, and 0.909 in Wave 9. Responses to each question were measured on a five-point Likert scale: the higher the score, the better the school adaptation. For school adaptation, we used the average values for the items measuring friendships, academics, and relationships with teachers.

Depressive symptoms were measured using ten items (Cronbach’s α: W7 = 0.918, W8 = 0.915, W9 = 0.933) and rated on a scale from 1 (*strongly disagree*) to 4 (*strongly agree*) per the depressive symptom scale of the Korean Mental Diagnosis Test (Kim et al., 1984). Social withdrawal was measured using five items (Cronbach’s α: W7 = 0.908, W8 = 0.905, W9 = 0.909) and rated on a scale from 1 (*strongly disagree*) to 4 (*strongly agree*) per the Social Withdrawal Scale (Lee et al., 2011). Self-esteem was measured with nine items (Cronbach’s α: W7 = 0.863, W8 = 0.883, W9 = 0.888) and rated on a scale from 1 (*strongly disagree*) to 5 (*strongly agree*) per Rosenberg’s Self-Esteem Scale (1965). For the analysis, we used the mean score of the items. A higher mean indicates higher depressive symptoms, social withdrawal, and self-esteem.

We used the Wave 7 data for the covariates—gender, monthly household income, and child neglect. Gender was coded as *male* = 0 and *female* = 1. Monthly household income was reported in increments of 100 thousand won^3^ from 0 to 10 million won, and we recoded it as follows: *990 thousand won or less* = 1, *between 1 million and 1.99 million won* = 2, *between 2 million and 2.99 million won* = 3, *between 3 million and 3.99 million won* = 4, and *up to and over 4 million won* = 5. Child neglect was measured using seven items (α = 0.821) from the Neglectful Parenting Scale (Huh, 2000), and all items were rated on a scale from 1 (*strongly disagree*) to 4 (*strongly agree*). The mean values of the items were used for analysis, and a higher mean indicates a higher level of child neglect. Area was coded as *rural area* = 0 and *urban area* = 1.

### Statistical Analysis

We utilized an autoregressive cross-lagged (ARCL) model to investigate the bidirectional long-term relationships between psycho-emotional states (depressive symptoms, social withdrawal, and self-esteem) and school adaptation. In this model, the autoregressive effect represents a coefficient derived by correlating the value of a variable at a given time (*t*) with its value at a preceding time (*t*–1). Additionally, the ARCL model calculated cross-lagged effects to assess the reciprocal impact of one variable at an earlier time (*t*–1) on a different variable at a subsequent time (*t*). We performed the analysis using AMOS version 23 software.

We first identified the autoregressive effects of multicultural adolescents’ depressive symptoms, social withdrawal, self-esteem, and school adaptation over time. Next, we investigated the cross-lagged effects of their psycho-emotional state (depressive symptoms, social withdrawal, and self-esteem) and school adaptation over time. Finally, we analyzed the impact of multicultural adolescents’ gender, household income, child neglect, and area on their depressive symptoms, social withdrawal, self-esteem, and school adaptation.

## Results

### Descriptive Statistics

Table 1 presents the descriptive statistics. The distributions of all variables were normal, with skewness values under three and kurtosis values under ten (Kline, 2011), which allowed us to proceed with the ARCL model.

**Table 1:** Descriptive Statistics.


VARIABLE	MEAN	*SD*	RANGE	SKEWNESS	KURTOSIS

Depressive symptoms W7	1.694	0.544	1–4	0.429	–0.342

Depressive symptoms W7	1.742	0.567	1–4	0.320	–0.498

Depressive symptoms W7	1.734	0.620	1–4	0.499	–0.343

Social withdrawal W7	2.365	0.729	1–4	–0.215	–0.439

Social withdrawal W8	2.384	0.718	1–4	–0.216	–0.368

Social withdrawal W9	2.340	0.723	1–4	–0.206	–0.525

Self-esteem W7	3.834	0.603	1–5	–0.175	–0.074

Self-esteem W8	3.828	0.663	1–5	–0.210	–0.027

Self-esteem W9	3.818	0.677	1–5	–0.283	0.268

School adaptation W7	3.768	0.578	1–5	–0.127	0.249

School adaptation W8	3.770	0.617	1–5	–0.311	0.903

School adaptation W9	3.825	0.614	1–5	–0.401	0.986

Gender W7	0.505	0.500	0, 1	–0.020	–2.006

Household income W7	3.335	1.060	1–5	–0.019	–0.902

Child neglect W7	1.758	0.510	1–4	0.333	–0.359

Area W7	0.652	0.477	0, 1	–0.638	–1.599

Age W7	15.960	0.319	–	–	–

Age W8	16.960	0.319	–	–	–

Age W9	17.960	0.319	–	–	–

	**Frequencies**	**%**	–	–	–

Gender W7					

Boys	294	49.5	–	–	–

Girls	300	50.5	–	–	–

Area W7					

Rural	207	34.8	–	–	–

Urban	387	65.2	–	–	–

Mother’s Nationality W7					

China	259	43.6	–	–	–

Vietnam	23	3.9	–	–	–

Philippine	269	45.3	–	–	–

Thailand	43	7.2	–	–	–

N	594				


*Note*. W7 = Wave 7; W8 = Wave 8; W9 = Wave 9.

### ARCL Model Estimation Results

Tables 2, 3, and 4 present the ARCL analysis results. First, the autoregressive effects of depressive symptoms, social withdrawal, self-esteem, and school adaptation were statistically significant across the three years. Depressive symptoms, social withdrawal, self-esteem, and school adaptation in Waves 7 and 8 positively affected the same variable in Waves 8 and 9, respectively, indicating that those who had reported high values for each variable in the past may have also reported high values for each variable later.

**Table 2:** Autoregressive Cross-lagged Effects between Depressive Symptoms and School Adaptation.


PATH	*β*	SE

Depressive symptoms W7 → Depressive symptoms W8	0.577***	0.035

Depressive symptoms W8 → Depressive symptoms W9	0.648***	0.036

School adaptation W7 → School adaptation W8	0.598***	0.035

School adaptation W8 → School adaptation W9	0.668***	0.03

Depressive symptoms W7 → School adaptation W8	–0.122***	0.037

Depressive symptoms W8 → School adaptation W9	–0.055	0.033

School adaptation W7 → Depressive symptoms W8	–0.069*	0.033

School adaptation W8 → Depressive symptoms W9	–0.038	0.034

Gender → Depressive symptoms W7	0.195***	0.04

Household income → Depressive symptoms W7	–0.02	0.019

Child neglect → Depressive symptoms W7	0.416***	0.039

Area → Depressive symptoms W7	0.121**	0.042

Gender → School adaptation W7	0.002	0.044

Household income → School adaptation W7	0.037	0.021

Child neglect → School adaptation W7	–0.388***	0.043

Area → School adaptation W7	–0.193***	0.047


*Note. N* = 594. Unstandardized beta coefficients reported. Model fit: *χ*^2^(23) = 276.253 (*p* < .001); GFI = 0.914; RMR = 0.035. W7 = Wave 7; W8 = Wave 8; W9 = Wave 9. *p < 0.05; **p < 0.01; ***p < 0.001. Area reference variable = rural area.

**Table 3:** Autoregressive Cross-lagged Effects between Social Withdrawal and School Adaptation.


PATH	*β*	SE

Social withdrawal W7 → Social withdrawal W8	0.564***	0.032

Social withdrawal W8 → Social withdrawal W9	0.655***	0.031

School adaptation W7 → School adaptation W8	0.651***	0.035

School adaptation W8 → School adaptation W9	0.677***	0.029

Social withdrawal W7 → School adaptation W8	–0.001	0.028

Social withdrawals W8 → School adaptation W9	–0.041	0.026

School adaptation W7 → Social withdrawal W8	–0.159***	0.04

School adaptation W8 → Social withdrawal W9	–0.083*	0.036

Gender → Social withdrawal W7	0.146*	0.059

Household income → Social withdrawal W7	–0.058*	0.028

Child neglect → Social withdrawal W7	0.194***	0.058

Area → Social withdrawal W7	0.02	0.062

Gender → School adaptation W7	0.002	0.044

Household income → School adaptation W7	0.037	0.021

Child neglect → School adaptation W7	–0.388***	0.043

Area → School adaptation W7	–0.193***	0.047


*Note. N* = 594. Unstandardized beta coefficients reported. Model fit: *χ*^2^(23) = 185.16 (*p* < .001); GFI = 0.943; RMR = 0.033. W7 = Wave 7; W8 = Wave 8; W9 = Wave 9. *p < 0.05; ***p < 0.001. Area reference variable = rural area.

**Table 4:** Autoregressive Cross-lagged Effects between Self-esteem and School Adaptation.


PATH	*β*	SE

Self-esteem W7 → Self-esteem W8	0.929***	0.024

Self-esteem W8 → Self-esteem W9	0.563***	0.036

School adaptation W7 → School adaptation W8	0.433***	0.032

School adaptation W8 → School adaptation W9	0.603***	0.034

Self-esteem W7 → School adaptation W8	0.41***	0.031

Self-esteem W8 → School adaptation W9	0.132***	0.03

School adaptation W7 → Self-esteem W8	0.011	0.025

School adaptation W8 → Self-esteem W9	0.139***	0.041

Gender → Self-esteem W7	–0.116**	0.043

Household income → Self-esteem W7	0.034	0.021

Child neglect → Self-esteem W7	–0.557***	0.042

Area → Self-esteem W7	–0.137**	0.046

Gender → School adaptation W7	0.002	0.044

Household income → School adaptation W7	0.037	0.021

Child neglect → School adaptation W7	–0.388***	0.043

Area → School adaptation W7	–0.193***	0.047


*Note. N* = 594. Unstandardized beta coefficients reported. Model fit: *χ*^2^(23) = 306.083 (*p* < 0.001); GFI = 0.911; RMR = 0.039. W7 = Wave 7; W8 = Wave 8; W9 = Wave 9. **p < 0.01; ***p < 0.001. Area reference variable = rural area.

Next, the cross-lagged analysis revealed reciprocal effects between depressive symptoms and school adaptation. Statistically significant coefficients were found on the paths from depressive symptoms in Wave 7 to school adaptation in Wave 8 (β = –0.122, *p* < .001) and from school adaptation in Wave 7 to depressive symptoms in Wave 8 (β = –0.069, *p* < .05). These results indicate that high depressive symptoms in multicultural adolescents in their first year of high school can lead to their school maladaptation in their second year. Multicultural adolescents who do not adjust well to school in the first year of high school may exhibit high levels of depressive symptoms in the second year.

In addition, the cross-lagged analysis of social withdrawal and school adaptation indicated that while social withdrawal did not affect school adaptation, school adaptation significantly affected social withdrawal. Statistically significant coefficients were found on the path from school adaptation in Waves 7 and 8 to social withdrawal in Waves 8 and 9, respectively (β = –0.159, *p* < .001; β = –0.083, *p* < .05), suggesting that multicultural adolescents with better school adaptation are less likely to be socially withdrawn during high school.

Finally, the cross-lagged analysis of self-esteem and school adaptation produced statistically significant coefficients on the paths from self-esteem in Waves 7 and 8 to school adaptation in Waves 8 and 9, respectively (β = 0.41 *p* < .001; β = 0.132, *p* < .001), and from school adaptation in Wave 8 to self-esteem in Wave 9 (β = 0.139, *p* < .001). These results suggest that the higher multicultural adolescents’ self-esteem during high school, the better they adjust to school. Multicultural adolescents who adapt well to school in the second year of high school may present higher self-esteem in the third year.

Other notable findings are as follows. In Wave 7, compared to boys, girls exhibited higher depressive symptoms (β = 0.195, *p* < .001) and social withdrawal (β = 0.146, *p* < .05) and lower self-esteem (β = –0.116, *p* < .01). Moreover, the higher the household income in Wave 7, the lower multicultural adolescents’ social withdrawal (β = –0.056, *p* < .05) in Wave 7. Child neglect in Wave 7 increased depressive symptoms (β = 0.417, *p* < .001) and social withdrawal (β = 0.194, *p* < .001) while lowering self-esteem (β = –0.558, *p* < .001) in Wave 7. Higher child neglect in Wave 7 was associated with poorer school adaptation among multicultural adolescents (β = –0.39, *p* < .01) in Wave 7. Multicultural adolescents in urban areas in Wave 7 exhibited higher levels of depressive symptoms (β = 0.121, *p* < .001), lower self-esteem (β = –0.137, *p* < .001), and poorer school adaptation (β = –0.193, *p* < .001) in Wave 7 compared to their peers in rural areas.

## Discussion

We examined the autoregressive and cross-lagged effects among depressive symptoms, social withdrawal, self-esteem, and school adaptation using longitudinal data on multicultural adolescents in Korea. First, our findings demonstrate positive autoregressive longitudinal effects for these variables, suggesting that the levels of depressive symptoms, social withdrawal, self-esteem, and school adaptation tend to persist over time. In other words, consistent with previous research (Heo & Kim, 2016; Lee et al., 2024; Lim, 2024; Tak et al., 2017), multicultural adolescents who reported high values for these variables in the previous year will likely report high levels for the same variables in the following year. The social and cultural environment generally influences psychosocial issues (Jang & Park, 2019; Mo, 2018; Rumbaut, 2005). Because these environmental factors often do not change significantly during the three years of high school, there may not be substantial changes in multicultural adolescents’ levels of depressive symptoms, social withdrawal, self-esteem, or school adaptation. These results suggest that depressive symptoms, social withdrawal, self-esteem, and school adaptation in multicultural adolescents may not be temporary but rather persist during high school.

Second, this study reveals bidirectional relationships between depressive symptoms and school adaptation in multicultural adolescents during their first and second years of high school. In other words, the higher the multicultural adolescents’ depressive symptoms, the more difficulty they had adapting to school. The more they failed to adapt to school, the more their depressive symptoms increased. These results partially support previous studies analyzing the relationship between depressive symptoms and school adaptation (Lin et al., 2011; Yu & Kim, 2018) which have suggested a unidirectional relationship between these variables, i.e., that depressive symptoms negatively affect school adaptation or that school maladaptation increases depressive symptoms. Still, our findings suggest that the relationship between these variables is reciprocal. Depressive symptoms can increase school maladaptation by reducing interest, motivation, and concentration in learning and school activities, which makes it difficult to build relationships with friends and teachers (Humensky et al., 2010). Additionally, multicultural adolescents who experience school maladaptation may receive negative feedback from teachers or peers, become stressed, and develop a negative self-perception, which can worsen depressive symptoms (Lin et al., 2008).

Third, school adaptation significantly affected social withdrawal, while social withdrawal did not predict future school adaptation. These results are consistent with previous studies showing that school adaptation reduces social withdrawal (Jo & Oh, 2020). By contrast, our findings challenge past studies that have concluded that social withdrawal negatively influences school adaptation (Bohlin et al., 2005). Overall, the results highlight the unidirectional relationship between these variables, demonstrating that school maladaptation rather increases the risk for social withdrawal than vice versa. Importantly, these results do not indicate that multicultural adolescents cannot adapt to school because they are socially withdrawn but rather that adolescents with pre-existing school adaptation problems are more likely to be socially withdrawn.

Even if multicultural adolescents are shy and passive, they may not have much difficulty adapting to school if they follow the rules and are committed to their school life. As the proportion of multicultural adolescents increases in Korea and their school adaptation becomes an important social issue, schools are helping them adapt to school by providing support programs and services such as mentoring programs, language support, and cultural exchange activities (Ministry of Gender Equality and Family, 2023). In addition, schools are conducting multicultural understanding education to help teachers and students develop an attitude of understanding and respect for cultural diversity. These measures can help reduce discrimination against and the exclusion of multicultural adolescents in schools (Min, 2009) and can contribute to the school adaptation of socially withdrawn multicultural adolescents.

Otherwise, when multicultural adolescents are unable to adapt to school, they may feel left out, experience lower confidence, and develop an increasingly negative perception of themselves, which can lead them to avoid social interaction and become socially withdrawn (Bowker et al., 2014). School adaptation—especially in terms of grades—is highly emphasized for high school teenagers, as Korean society places great importance on academic achievement. Because adolescents are evaluated primarily on the basis of their grades, adolescents with poor grades may have difficulties in school adaptation overall, which may lead to social withdrawal. Good grades or friendships at school can be a more significant psychological reward for multicultural adolescents because they tend to have a lower socioeconomic status than non-multicultural adolescents and lack social networks such as family, relatives, and acquaintances.

Fourth, while the self-esteem of first-year high school multicultural adolescents positively affected their second-year school adaptation, which is consistent with previous research (Jeon & Yoon, 2013; Kim, 2019), school adaptation did not affect their self-esteem, which is inconsistent with previous research (Chun, 2018; Kim, 2012; Lee & Lee, 2019). However, self-esteem and school adaptation had bidirectional relationships in these adolescents’ second and third years of high school. In other words, the higher their self-esteem in the second year of high school, the better their school adaptation in the third year, which supports previous research (Jeon & Yoon, 2013; Kim, 2019). The better multicultural adolescents adjust to school in their sophomore year, the higher their self-esteem in their third year, which is also consistent with previous research (Chun, 2018; Kim, 2012; Lee & Lee, 2019). Indeed, in high school, the importance of study and academic stress increases with each year. Therefore, school adaptation may have affected these adolescents’ self-esteem in the second year, unlike in the first year.

Previous studies have also demonstrated that depressive symptoms and social withdrawal are higher and self-esteem is lower in female versus male multicultural adolescents (Mo, 2018; Oh, 2016). Among multicultural adolescents, like other adolescents, girls may exhibit more depression than boys due to hormonal changes (Essau et al, 2010). Girls may be more relationship-oriented than boys, compare themselves to others more frequently, and be more sensitive to others’ evaluations (Girgus & Yang, 2015). These tendencies could make girls feel more socially withdrawn compared to boys. Also, gender differences in social withdrawal may reflect differences in social acceptability, as the prominent emotional features of withdrawal—such as fear, anxiety, and shyness—are considered feminine by society (Rubin & Barstead, 2014). In society, boys’ expressions of emotions such as sadness, fear, anxiety, shame, and guilt are often regarded as signs of weakness, and they are expected to suppress them (Naito et al., 2005). In this context, girls may be more likely to report feelings of social withdrawal given the greater social acceptability of such emotions for girls than for boys. For instance, even if a boy and a girl experience the same feelings of withdrawal, the girl might acknowledge such feelings and respond honestly on a questionnaire. However, the boy may not be accustomed to openly admitting such emotions.

In adolescence, girls experience more dissatisfaction with their body image compared to boys, which is found to contribute to depression and low self-esteem (Van den Berg et al., 2002). Girls also tend to be more concerned about pro-social activities and their general acceptance by others. If girls feel that certain social activities are important but that they are not good at them, they may experience more negative effects on their self-esteem than boys (Bhamani et al., 2014).

Consistent with previous research (Damelang & Kloß, 2013), low household income was associated with increased social withdrawal in multicultural adolescents. In addition to economic constraints on participating in social activities such as organized sports, music, and cultural activities with peers, the shame that multicultural adolescents feel about poverty can lead them to be socially withdrawn (Damelang & Kloß, 2013). Moreover, our results are consistent with existing studies showing that child neglect negatively affects adolescents’ psychosocial development (Choe, 2021; Oshri et al., 2017). In other words, the deficiencies and dissatisfaction resulting from not meeting basic needs such as adequate nutrition, emotional support, hygiene, health care, cognitive stimulation, and safe living conditions can lead adolescents to become socially withdrawn and depressed (Stoltenborgh et al., 2013; Wang et al., 2023).

Adolescents who are neglected by their parents do not receive enough attention or care to develop a sense of self-worth. They internalize the message of their worthlessness and assume that they will not succeed in acquiring friends, achieving school success, or being noticed (Egeland et al., 2002). Negative perceptions about themselves and others, negative interpersonal expectations, and difficulties in resolving interpersonal problems can make it difficult to build relationships with friends and teachers at school and interfere with their school adaptation overall (Choe, 2021).

Regarding area of residence, in line with previous studies (Dobbins et al., 2022; Elgar et al., 2003; Yilmaz et al., 2022), our results reveal that multicultural adolescents in rural areas exhibit better psychosocial states in terms of depression, self-esteem, and school adaptation than those in urban areas. This may be because in rural areas, relationships between neighbors are closer and social support is more accessible, enabling multicultural adolescents to feel less lonely and have their cultural identity recognized (Biggar et al., 2016). Rural schools also have fewer students, facilitating more frequent interactions between teachers and students as well as personalized education, which may help students better adapt to school and experience less academic stress. Additionally, the natural environment of rural areas can contribute to stress reduction and emotional stability in multicultural adolescents. Compared to rural areas, cities have larger populations, resulting in relatively superficial relationships between people, fierce academic and social competition, and significant economic disparities (Elgar et al., 2003). Therefore, this urban environment may negatively affect the psychosocial adaptation of multicultural adolescents.

### Implications

During adolescence, significant physical and mental changes occur and parental expectations and social demands increase, making adolescents vulnerable to psychosocial problems such as depressive symptoms, social withdrawal, low self-esteem, and school maladaptation (Kim, 2011). Due to the intense academic pressure and fierce competition for college entrance exams, these problems tend to increase as Korean adolescents grow older (An et al., 2013; Jeon et al., 2019). In general, adolescents in Korea face challenges such as depressive symptoms, social withdrawal, low self-esteem, and school maladaptation. However, multicultural adolescents are more vulnerable to these problems than non-multicultural adolescents because they experience cultural conflicts, racial discrimination, a lack of academic support, and economic difficulties (Chae, 2018; Jang & Park, 2019).

The autoregressive effects of variables imply that once multicultural adolescents’ depressive symptoms, social withdrawal, low self-esteem, and school maladaptation appear, they can easily continue or even worsen during high school. Therefore, when these adolescents experience such problems, parents and practitioners should not perceive this phenomenon as necessarily temporary. Instead, these students require early intervention and help with the ongoing management of those problems.

In other countries, it has been emphasized that immigrant children’s school maladaptation negatively impacts their mental health (Shoshani et al., 2015; Xie et al., 2022). However, this study suggests that not only does school maladaptation among Korean multicultural adolescents increase their depressive symptoms, but depressive symptoms can also increase their school maladaptation. Like immigrant children in other countries, high self-esteem has been shown to positively affect school adaptation among these adolescents (Bankston & Zhou, 2002; Carranza et al., 2009). For multicultural adolescents in Korea, depressive symptoms and school maladaptation in the second year of high school can be prevented through interventions targeting these factors in the first year. Moreover, to improve multicultural adolescents’ school adaptation during high school, it is crucial to improve their self-esteem. To do so, practitioners must examine the parenting styles of the parents of these adolescents. For example, at the beginning of students’ first year of high school, schools can survey them about their parents’ parenting attitudes or send parents a link to a parenting attitude survey and have them complete it. Then, improving parenting attitudes through counseling or education at schools or family centers for those parents who exhibit neglectful parenting attitudes as identified by the survey can help alleviate multicultural adolescents’ depressive symptoms and improve their self-esteem and school adaptation.

In Korea, family centers support the psychological and emotional stability of multicultural adolescents by providing services such as parent education, parent–child relationship improvement programs, and psychological counseling (Ministry of Gender Equality and Family, 2023). However, these programs are mainly aimed at elementary school students, and programs targeting high school students are lacking.

Additionally, there are few psycho-emotional support programs that focus on child neglect. Neglectful parents rarely communicate with their children, lack intimacy, and are emotionally distant (Biçakçi et al., 2016). It is crucial to help parents become closer to their children through activities or play programs that parents and children can do together and improve communication skills that allow parents to accept, respect, and respond to their children’s needs and feelings. For parents who are unable to fully meet their children’s needs due to a lack of economic resources, cooperation should be established with child welfare centers, family support centers, and social welfare centers to ensure these parents can provide the necessary resources for their children (Avdibegovie & Brkie, 2020). Parents with stress, depression, and anxiety may not be able to afford to take care of themselves, making it difficult to give attention and affection to their children (Schumacher et al., 2001). Therefore, providing stress management programs and psychological support to parents with psychological difficulties so that they can resolve their mental problems can contribute to reducing child neglect.

Foreign mothers of multicultural adolescents are unfamiliar with Korean culture and language. As they lack educational information, they are often not deeply involved in their children’s education and readily excluded from decision-making about their children’s work (Choi & Hong, 2017). As a result, foreign mothers may not be able to understand or fully satisfy their children’s various needs in high school, which may lead to child neglect. Therefore, educational materials translated into Chinese, Vietnamese, Thai, and English need to be produced and placed in schools and welfare facilities to support foreign mothers.

Unlike immigrant children in other countries, whose social withdrawal has been shown to negatively impact their school adaptation (Balkaya et al., 2017; Iannello et al., 2024), Korean multicultural adolescents exhibit increased social withdrawal due to school maladaptation. Therefore, assisting multicultural adolescents in Korea with school adaptation can help reduce their social withdrawal. In particular, helping sophomore-year multicultural adolescents solve problems relating to school adaptation, especially regarding school study, can alleviate their social withdrawal. As mentioned above, school adaptation can also be improved by reducing the neglect of these children by their parents.

In addition, providing financial support for multicultural adolescents in their learning can help reduce their social withdrawal. The household income of multicultural families tends to be lower than that of non-multicultural families (Kim, 2022). Accordingly, due to insufficient household income for their learning (e.g., private education, study materials, and leisure), high school multicultural adolescents may be limited in terms of social and learning activities and expenses for socializing with Korean friends. To address this issue, the government provides learning support to multicultural adolescents through after-school academies that offer assistance with homework, supplementary study, and reading guidance for those with learning difficulties (Ministry of Gender Equality and Family, 2023). In addition, the government need to help these students by operating programs to subsidize their tuition or academic activity expenses, given that multicultural adolescents often experience poor economic conditions, a lack of learning and social support, and lower academic achievement compared to their non-multicultural peers (Baik et al., 2014; Kim, 2022). These measures can help revitalize these students’ social participation and address their academic challenges, thereby helping reduce their school maladaptation and social withdrawal.

### Limitations and Future Directions

One strength of this study is that it investigates how to support adolescent groups that are vulnerable to psychosocial problems due to cultural conflict, discrimination, and a lack of social support. Nevertheless, this study faces certain limitations. This study exclusively considers adolescents from multicultural families in Korea, which predominantly comprise Korean men and foreign women. In Korea, multicultural families, predominantly comprising Korean men and foreign women, represent a significant portion of international marriages and possess distinct characteristics. These include situations like low-income or older Korean men marrying younger women from less developed countries (such as China, Southeast Asia, and Central Asia), marriages facilitated by international brokers over a short term, and situations where the husband bears all marriage-related expenses. These specific forms of international marriages in Korea may differ significantly from those in other countries, which limits the applicability of our findings on a global scale.

Additionally, while Korean multicultural families predominantly consist of Korean men and foreign women, they can also comprise Korean women and foreign men. Such families primarily consist of men from the United States or Europe and Korean women. Compared to the former group, these families are economically better off and experience less discrimination in Korea. Additionally, their children often attend private international schools, and even when attending Korean public schools, they generally face fewer difficulties in adaptation than the former group.

Moreover, although this study examined adolescents born in Korea, some adolescents were born overseas and later relocated to Korea. Consequently, our findings may not fully apply to these types of multicultural adolescents. Future research should, therefore, aim to explore the psychosocial challenges faced by multicultural adolescents considering a more diverse array of backgrounds and experiences. In addition, because the data used in this study were collected using self-report measures rather than structured diagnostic interviews, they could be subject to self-report bias, potentially inflating the associations between variables. Future research should include objective measures to improve the reliability and validity of the data.

## Additional File

The additional file for this article can be found as follows:

10.5334/pb.1310.s1 Supplementary Material. Measurement items.

## Footnotes

Democratic parenting behavior is a balanced approach to childrearing that combines high expectations with responsiveness and support. It is characterized by open communication, encouraging children to make their own choices and decisions, establishing clear rules and guidelines for behavior, parental warmth and nurturance, and a balance between maintaining authority and granting freedom (Basumatary & Baglari, 2021; Baumrid, 1991).

Families with foreign women from less developed countries and Korean men frequently fall into the categories of ‘low-income’ and ‘vulnerable’ in Korean society.

As of July 15, 2024, 1 dollar and 1 euro are equivalent to 1,377 won and 1,502 won, respectively.
