# Supplementary material for: Longitudinal Cross-Lagged Analysis Between Depressive Symptoms, Social Withdrawal, Self-Esteem, and School Adaptation in Multicultural Adolescents
Source: Psychol Belg. 2025 Jan 20;65(1):38–53. doi: 10.5334/pb.1310 (PMC11760225; doi:10.5334/pb.1310)
Supplement: Supplementary Material. — Measurement items. [file pb-65-1-1310-s1.pdf]

SUPPLEMENTARY MATERIAL

Measurement items

Variable	Item
Depressive symptoms	Q1. I don't have much energy.<br>Q2. I feel unhappy, sad or depressed.<br>Q3. I have a lot of worries.<br>Q4. I feel like dying.<br>Q5. I'm good at crying.<br>Q6. I often feel like it's my fault when something goes wrong.<br>Q7. I am lonely.<br>Q8. I have no interest or enthusiasm for anything.<br>Q9. I don't feel hopeful about the future.<br>Q10. Everything is hard for me.
Social withdrawal	Q1. I feel awkward when there are a lot of people around me.<br>Q2. I am very shy.<br>Q3. I have difficulty expressing my opinions clearly to others.<br>Q4. I have pudency.<br>Q5. I hate being in front of people.
Self-esteem	Q1. I think I am as valuable as anyone else.<br>Q2. I think I have good character.<br>Q3. I think, overall, I am a failure. (reverse-coded)<br>Q4. I can do things as well as most other people.<br>Q5. I don't have much to brag about. (reverse-coded)<br>Q6. I have a positive attitude about myself.<br>Q7. I am generally satisfied with myself.<br>Q8. Sometimes I feel like I'm worthless. (reverse-coded)<br>Q9. Sometimes I think I'm not a good person. (reverse-coded)
Gender	Respondent's gender (male, female)
Household income	What is your average monthly household income level?
Child neglect	Q1. My parents (guardians) seem to think outside work is more important than me.<br>Q2. My parents (guardians) are not interested in me, so they never praise or scold me.<br>Q3. My parents (guardians) are not interested in what I think.<br>Q4. My parents (guardians) are not there when I need them.<br>Q5. Even when I'm sick, my parents (guardians) don't bother to take me to the hospital.<br>Q6. My parents (guardians) are interested and ask how I am doing at school. (reverse-coded)<br>Q7. My parents (guardians) always take care to keep my body, clothes, and bedding clean. (reverse-coded).
Area	Areas where respondent lives (rural areas, urban areas)
